# Detection of the GH Analogue Redalsomatropin Alfa in Sports Drug Testing: Immunological Approaches and LC‐HRMS/MS

**DOI:** 10.1002/dta.70075

**Published:** 2026-04-21

**Authors:** Katja Walpurgis, Andreas Thomas, Jasmin Thelen, Bettina Majer, Maneera Al‐Jaber, Wadha Abushareeda, Mario Thevis

**Affiliations:** ^1^ Institute of Biochemistry/Center for Preventive Doping Research German Sport University Cologne Cologne Germany; ^2^ Anti‐Doping Lab Qatar Doha Qatar; ^3^ European Monitoring Center for Emerging Doping Agents (EuMoCEDA) Cologne/Bonn Germany

**Keywords:** affinity purification, doping, LC‐HRMS/MS, long‐acting growth hormone (LAGH), tryptic digestion

## Abstract

Human growth hormone (hGH) is a peptide hormone exerting different growth‐promoting and metabolic functions through binding to the GH receptor (GHR). Due to the presumed lipolytic and anabolic effects, the misuse of recombinant 22‐kDa hGH in sports is prohibited both in‐ and out‐of‐competition, and also synthetic long‐acting GH analogues were added to the WADA Prohibited List in 2022. Within this research project, the detectability of redalsomatropin alfa (JR‐142) with the immunoassays routinely employed by anti‐doping laboratories for rhGH testing was evaluated. The drug candidate represents a recombinant fusion protein of 22‐kDa hGH (191 amino acids) and human serum albumin (HSA, 585 amino acids) and is currently undergoing Phase III of its clinical development. Due to the attachment of HSA to the C‐terminus of 22‐kDa hGH, only “Kit 2” of the routinely employed immunoassay was found to recognize the drug, and the cross‐reactivity was significantly reduced when compared to native hGH. As the misuse of redalsomatropin alfa in sports can therefore remain undetected when using this assay alone, the drug was implemented into the existing qualitative initial testing procedure (ITP) for the hGH analogue somatrogon employing affinity purification with GHR‐Fc‐conjugated magnetic beads for sample extraction and tryptic digestion in combination with LC‐HRMS/MS for protein identification. Method validation demonstrated that the assay allows for a sensitive and specific detection of the fusion protein down to concentrations of 50 ng/mL. Moreover, these findings show that this LC‐HRMS/MS assay can be further expanded to simultaneously test for multiple long‐acting growth hormones (LAGHs).

## Graphical abstract

After evaluating the compatibility of the GH analogue redalsomatropin alfa with approved doping control immunoassays for rhGH, it was implemented into the existing detection method for somatrogon employing affinity purification with GHR‐Fc‐conjugated magnetic beads, tryptic digestion, and LC‐HRMS/MS to allow for a specific and sensitive detection.

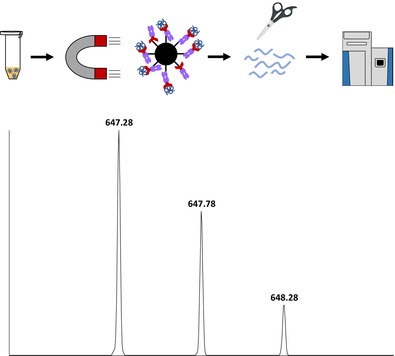

## 1 Introduction

Since the market introduction of recombinant human growth hormone (rhGH) in the 1980s, it represents not only an important protein therapeutic for the treatment of GH deficiency in both children and adults but also a potential performance‐enhancing agent in sports [1, 2]. Due to the presumed growth‐promoting and metabolic effects, its misuse both in‐ and out‐of‐competition was prohibited by the World Anti‐Doping Agency (WADA) shortly thereafter, and two detection strategies comprising the quantitative determination of pituitary GH isoforms with different immunoassays (“hGH isoform differential immunoassays”) and the analysis of GH‐dependent biomarkers (“hGH biomarkers test”) were established.

**FIGURE 1:**
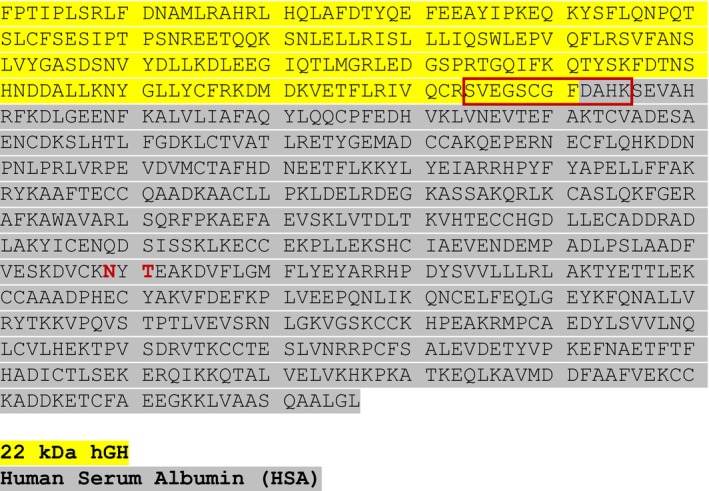
Amino acid sequence of redalsomatropin alfa. The amino acids of 22‐kDa hGH are highlighted in yellow, and those of HSA in grey. Red letters indicate amino acid exchanges/modifications, and the diagnostic peptide T_21_ is encircled in red.

Due to the high injection frequency required to ensure rhGH efficacy in therapeutic settings, several strategies for the development of long‐acting growth hormone (LAGH) preparations were tested during the last decades, comprising depot, PEGylated, and prodrug formulations as well as modified rhGH variants. Albumin binder conjugates such as somapacitan (Sogroya, Novo Nordisk) strongly bind to human serum albumin (HSA) via a special binding moiety. By contrast, hGH fusion proteins are characterized by an increased molecular weight (MW) and, therefore, reduced renal clearance [3]. Whereas somatrogon (OPKO Health and Pfizer) is a fusion protein of rhGH and three copies of the C‐terminal peptide of the β‐subunit of human chorionic gonadotropin (hCG), rhGH is fused to immunoglobulin fragments in AG‐B1512 (Ahngook Pharmaceutical), GX‐H9 (Genexine and Handok), and LAPS rhGH (Hanmi Pharmaceutical). Other constructs were developed with a recombinant a1‐antitrypsin derivative, a GH binding protein, and nonsense amino acid sequences as fusion partners. Redalsomatropin alfa, also known as JR‐142, is a recombinant fusion protein where modified HSA (585 amino acids) is attached to the C‐terminus of 22‐kDa hGH (191 amino acids, Figure 1), resulting in a total MW of approximately 88 kDa [4]. Compared to native HSA, the asparagine residue at position 509 is N‐glycosylated, and the alanine residue at position 511 is replaced with a threonine. While the increased MW of redalsomatropin alfa results in a reduced renal elimination, the decreased affinity for the GHR leads to a lower GHR‐dependent clearance. Moreover, the attachment of 22‐kDa hGH to HSA further prolongs the biological half‐life as the fusion protein is recycled via the neonatal Fc receptor FcRn. Redalsomatropin alfa is currently undergoing Phase III clinical trials for the therapy of pediatric GH deficiency in Japan [5]. The development of a similar fusion protein where HSA is linked to the N‐terminus of 22‐kDa hGH (albutropin, TV‐1106) was discontinued after Phase II testing due to the production of neutralizing antidrug antibodies in several patients, which was—so far—not observed for redalsomatropin alfa [4, 6].

In 2022, also LAGHs were added to the WADA Prohibited List [7], but unfortunately, the immunoassays routinely employed by anti‐doping laboratories for GH testing [2] were found to be partially incompatible with the detection of GH analogues such as somatrogon [8]. As the C‐terminal epitope of 22‐kDa hGH [9] is concealed by the attached hCG‐β CTP, the capture antibody of “Kit 1” was found to have no cross‐reactivity with the fusion protein. Due to the large HSA molecule fused with the C‐terminus of redalsomatropin alfa, it can be expected that its detectability with the GH isoform test will also be impaired. Therefore, the aim of this research project was to investigate the compatibility of redalsomatropin alfa with the WADA‐approved doping control immunoassays for rhGH and implement the drug into the existing qualitative initial testing procedure (ITP) for somatrogon employing affinity purification with magnetic beads coupled to the GH receptor (GHR), tryptic digestion, and liquid chromatography high‐resolution tandem mass spectrometry (LC‐HRMS/MS) [8].

## 2 Material and Methods

### 2.1 Reference Material, Chemicals, and Consumables

Reference material for redalsomatropin alfa (JR‐142) was obtained from ProteoGenix (Schiltigheim, France). U‐^15^N‐labeled 22 kDa GH from Novo Nordisk (Bagsværd, Denmark) and kindly provided by Cristian Arsene (Physikalisch‐Technische Bundesanstalt, Braunschweig, Germany) was employed as internal standard (ISTD). NHS Magnetic Sepharose was purchased from Cytiva (Marlborough, MA) and coupled to a recombinant human GHR‐Fc chimera bought from R&D Systems (Minneapolis, MN). Dithiothreitol (DTT, as 1 M solution) and iodoacetamide (IAA) were used for protein reduction and alkylation and obtained from Merck (Darmstadt, Germany) and Thermo Fisher Scientific (Waltham, MA). Sequencing grade modified trypsin (V5111) was from Promega (Madison, WI). All other chemicals/solvents were from Merck (Darmstadt, Germany) and of analytical grade. For hGH isoform testing, immunoassays were bought from CMZ assay (Berlin, Germany).

### 2.2 Samples

Method validation was conducted by using human serum obtained from male AB plasma from Merck (Darmstadt, Germany). Additionally, both serum and plasma samples were collected from 10 healthy volunteers (five males and five females) following approval of the local ethical committee (DSHS No. 139/2021) and written informed consent.

Moreover, a total of 20 doping control routine serum samples (10 males and 10 females) tested negative in all analytical procedures were employed to investigate the detectability of redalsomatropin alfa with the GH isoform differential immunoassays.

### 2.3 GH Isoform Differential Immunoassays

#### 2.3.1 Cross‐Reactivity Test

The cross‐reactivity of redalsomatropin alfa with the GH isoform differential immunoassays routinely employed by anti‐doping laboratories [9, 10] was investigated by analyzing sheep serum fortified with the fusion protein at concentrations of 3, 30, and 300 ng/mL in duplicate with both Kits 1 and 2 according to the manufacturer's instructions. Luminescence measurements were carried out with an Auto Lumat Plus LB953 luminometer from Berthold Technologies GmbH (Bad Wildbad, Germany).

#### 2.3.2 Analysis of Fortified Serum Samples

A total of 20 serum samples tested negative for rhGH doping in the Cologne anti‐doping laboratory were fortified with redalsomatropin alfa at concentrations of 3 (*n* = 10), 30 (*n* = 10), and 300 ng/mL (*n* = 20) and analyzed in duplicate with GH isoform Kits 1 and 2 according to the instructions of the manufacturer on an Auto Lumat Plus LB953 luminometer. The resulting concentrations of rhGH and pituitary hGH (phGH) were used to calculate the Rec/Pit ratios to be compared to WADA thresholds [10].

### 2.4 Mass Spectrometric Detection of Redalsomatropin Alfa

#### 2.4.1 Identification of Target Peptides

The amino acid sequence of redalsomatropin (Figure 1) was obtained from an online database [11] and subjected to *in silico* tryptic digestion with GPMAW Software (Version 8.0, Lighthouse Data, Denmark) to identify peptides comprising either sequences of both hGH and HSA or amino acid exchanges. Then, a Protein BLAST database search [12] was conducted with the amino acid sequences of the resulting peptides T_21_ and T_63_ to ensure that they do not naturally occur in any other human protein. In parallel, in‐solution tryptic digestion was carried out with 100 ng of redalsomatropin alfa reference material following protein reduction and alkylation, and the digest was analyzed by means of LC‐HRMS/MS comprising FullMS and TopN data‐dependent MS/MS experiments (details not shown). For MS data evaluation, Thermo Xcalibur Software (Version 4.0.27.10, 2015) was used.

#### 2.4.2 Magnetic Bead Preparation

For method validation, 10 batches of GHR‐Fc‐conjugated magnetic beads were prepared as described in earlier studies [8, 13] according to the manufacturer's instructions. In brief, 25 μL of the medium slurry were transferred to an Eppendorf tube, and the storage solution was removed with a magnetic rack. Following equilibration with 500 μL of ice‐cold 1 mM HCl, the magnetic beads were mixed with 5 μg of GHR‐Fc diluted in 50 μL of coupling buffer (0.2 M NaHCO_3_, 0.5 M NaCl, pH 8.3) and incubated for 30 min at 1200 rpm and room temperature (RT). The supernatant was removed with a magnetic rack, and the beads were consecutively washed with 500 μL of blocking buffer A (50 mM Tris–HCl, 1 M NaCl, pH 8.0) and blocking buffer B (50 mM glycine, 1 M NaCl, pH 3.0) for a total of three times. Here, the second washing step with blocking buffer A was carried out for 15 min on a rotating sample mixer. After equilibrating the beads twice in 500 μL of phosphate‐buffered saline (PBS; 1 tablet per 200 mL of deionized water: 0.01 M phosphate buffer, 0.0027 M potassium chloride and 0.137 M sodium chloride, pH 7.4), they were finally reconstituted with 300 μL of PBS, pooled, and stored at 4°C until usage.

#### 2.4.3 Sample Extraction

Of each sample, 100 μL of serum/plasma were aliquoted, fortified with 25 ng of ISTD (5 μL of a solution with 5 μg/mL), and diluted with 100 μL of PBS buffer. Then, they were mixed with 300 μL of GHR‐Fc‐conjugated magnetic beads each and incubated for approximately 60 min at RT on a rotating sample mixer. Magnetic beads were washed with 500 μL of PBST (0.02% Tween 20) once and 500 μL of PBS twice, and ligands bound to the GHR were eluted with 50 μL of 3% acetic acid (HAc) for 15 min at 1200 rpm and RT. Eluates were transferred to fresh Protein LoBind tubes, and magnetic beads were washed with 300 μL of 3% HAc (3×), 500 μL of PBST (1×), and 500 μL of PBS (2×). For reusage, they were finally resuspended in 300 μL of PBS and returned to the magnetic bead stock solution stored at 4°C.

#### 2.4.4 Tryptic Digestion

Neutralization of the acidic sample extracts was accomplished with 25 μL of 2 M NH_4_HCO_3_. Then, they were subjected to protein reduction with 750 nmol of DTT (7.5 μL of a 0.1 M solution in 100 mM NH_4_HCO_3_) for 30 min at 60°C and 900 rpm and alkylation with 1875 nmol of iodoacetamide (7.5 μL of a 0.25 M solution in 100 mM NH_4_HCO_3_) for 30 min at RT in the dark. For proteolysis, 400 ng of trypsin (10 μL of a solution with 40 μg/mL in 50 mM NH_4_HCO_3_) and 10 μL of acetonitrile (ACN) were added, and samples incubated overnight at 37°C and 500 rpm. After stopping tryptic digestion with 5 μL of glacial acetic acid, samples were transferred to polypropylene HPLC vials.

#### 2.4.5 LC‐HRMS/MS

As published elsewhere [8], LC‐HRMS/MS analysis was conducted on a Vanquish UHPLC coupled to an Orbitrap Exploris 480 mass spectrometer (Thermo Fisher Scientific). The LC system was equipped with an Accucore Phenyl‐Hexyl trapping column (3 × 10 mm, 2.6 μm; Thermo Fisher Scientific) and a Poroshell EC‐C18 analytical column (3 × 50 mm, 2.7 μm; Agilent Technologies, Santa Clara, CA, USA), which were both operated at a temperature of 30°C. The autosampler temperature was set to 10°C, and the injection volume to 10 μL. While 0.1% formic acid with 1% dimethyl sulfoxide (DMSO) in water was used as solvent A, 0.1% formic acid with 1% DMSO in ACN was employed as solvent B. The following LC gradient was run with a flow rate of 0.4 mL/min: 2 min of trapping with 5% B were followed by an increase in solvent B to 40% over 6 min and 80% over another 2 min. Then, the system was re‐equilibrated with 5% B for 5 min.

The mass spectrometer was operated in positive ionization mode with an ionization voltage of 4 kV. The temperature of the ion transfer tube was set to 320°C. Data were acquired in targeted selected ion monitoring (tSIM) mode with an isolation window of *m/z* = 3, a resolution of 60,000 FWHM at *m/z* 200, and an inclusion list with the accurate mass‐to‐charge ratios of the most abundant charge states of the target peptides for redalsomatropin alfa, somatrogon, 22‐kDa hGH and the ISTD (Table 1). Data‐dependent MS/MS (ddM^2^) experiments were conducted with a mass isolation window of *m/z* = 2, a first mass‐to‐charge ratio of *m/z* = 200, an intensity threshold of 1.0E4, a resolution of 15,000 FWHM at *m/z* 200, and a normalized collision energy (NCE) of 30%. Nitrogen obtained from a N_2_‐generator (CMC, Eschborn, Germany) was used as collision gas. Additionally, full‐scan spectra from *m/z* 400–2400 were recorded with a resolution of 30,000 full width at half maximum (FWHM) at *m/z* 200.

**TABLE 1:** Target peptides for LC‐HRMS/MS.

Analyte	Tryptic peptide #	Amino acid sequence	Amino acid positions	Modification(s)	*m/z* ^a^	Charge state
Redalsomatropin alfa	T_21_	SVEGSCGFDAHK	184–195	—	647.28	2
Somatrogon	T_1_–T_3_	SSSSKAPPPSLPSPSRLPGPSDTPILPQFPTIPLSR	1–36	4 × NANA‐Gal‐GalNAc	1584.99	4
				4 × NANA‐Gal‐GalNAc + HP	1588.99	4
				5 × NANA‐Gal‐GalNAc	1749.04	4
				5 × NANA‐Gal‐GalNAc + HP	1753.04	4
	T_3_	LPGPSDTPILPQFPTIPLSR	17–36	—	1074.10	2
	T_23_	SVEGSCGFSSSSK	212–224	—	659.78	2
22‐kDa hGH	T_2_	LFDNAMLR	9–16	—	490.26	2
	T_4_	LHQLAFDTYQEFEEAYIPK	20–38	—	1171.57	2
	T_10_	SVFANSLVYGASDSNVYDLLK	95–115	—	1131.57	2
U‐^15^N‐22‐kDa hGH (ISTD)	T_1_	FPTIPLSR	1–8	U‐^15^N‐labeling	471.26	2
	T_4_	LHQLAFDTYQEFEEAYIPK	20–38	U‐^15^N‐labeling	789.36	3
	T_9_	ISLLLIQSWLEPVQFLR	78–94	U‐^15^N‐labeling	1039.57	2

^a^ Most abundant isotopes, cysteine residues alkylated with iodoacetamide.

The MS was calibrated weekly according to the manufacturer's recommendations with a mixture of caffeine, the tetrapeptide MRFA (methionine–arginine–phenylalanine–alanine), and Ultramark 1621 (Thermo Fisher Scientific). For MS data evaluation, Thermo Xcalibur Software (Version 4.0.27.10, 2015) was employed.

#### 2.4.6 Method Validation

Method validation was conducted according to current WADA guidelines [14] applicable to qualitative ITPs, and the following parameters were determined for the target peptide T_21_:

- Selectivity

A total of 10 blank serum samples were analyzed and evaluated with regard to the presence of interfering signals at the retention time (RT) of the target peptide.

- Limit of detection (LOD)

To estimate the LOD, a total of six serum samples were fortified with redalsomatropin alfa at concentrations of 25, 50, and 75 ng/mL and prepared as described above. For each concentration, the detectability of T_21_ was evaluated, and a detection rate of > 95% was applied to determine the LOD.

- Reliability

The reliability was investigated by analyzing 10 different serum samples fortified with 100 ng/mL of redalsomatropin alfa.

- Robustness

The method's robustness was evaluated by using plasma instead of serum as biological matrix: Ten different plasma samples were fortified with 100 ng/mL of redalsomatropin alfa and analyzed as described above.

- Linearity

Linearity was assessed by analyzing serum samples fortified with 0, 50, 75, 100, 250, 500, 750, and 1000 ng/mL of redalsomatropin alfa. Absolute peak areas were used to construct a calibration curve, and a regression analysis was conducted.

- Carryover (LC‐HRMS/MS)
  The risk for sample carryover during LC‐HRMS/MS measurements was determined by injecting a blank serum extract immediately after the extract of a sample fortified with 1000 ng/mL of redalsomatropin alfa.
- Carryover (magnetic beads)
  To allow for reusage of the GHR‐Fc‐conjugated magnetic beads, the risk for carryover had to be investigated. For that purpose, a serum sample fortified with 1000 ng/mL of redalsomatropin alfa was eluted with 50 μL of 3% HAc and subsequently washed with 300 μL of 3% HAc for a total of three times. Of each wash fraction, 50 μL were subjected to neutralization, protein reduction and alkylation, tryptic digestion, and LC‐HRMS/MS analysis as described above.
- Stability
  Analyte stability was determined by reanalyzing the linearity sample extracts after 5 days of storage in the autosampler of the LC‐HRMS system at a temperature of 10°C.
- Recovery (magnetic beads)
  To determine the recovery of the GHR‐Fc‐conjugated magnetic beads, four blank serum specimens and four samples fortified with 25 ng of redalsomatropin alfa (final concentration: 250 ng/mL) were subjected to affinity purification, and the resulting blank sample eluates were also mixed with 25 ng of the drug. All samples were then processed and analyzed as described above. Finally, the recovery was estimated by comparing the absolute peak areas of the samples fortified with redalsomatropin alfa before and following extraction.

## 3 Results and Discussion

### 3.1 Detectability of Redalsomatropin Alfa With the GH Isoform Differential Immunoassays

In sports drug testing, two different approaches are routinely used for GH testing [2]: The biomarkers test is based on the quantification of the hGH‐dependent biomarkers insulin‐like growth factor I (IGF‐I) and P‐III‐NP (N‐terminal extension peptide of procollagen type III) in serum by using either immunoassays or LC‐HRMS/MS. Then, the GH‐2000 score is calculated with a gender‐specific discriminant function and compared to gender‐ and assay‐specific decision limits. The second approach employs two different kits (“1” and “2”) with two sandwich‐type chemiluminescence immunoassays each to determine the serum concentrations of 22‐kDa hGH/rhGH (“Rec”) alone and all pituitary hGH isoforms (phGH, “Pit”) [2, 9]. As the exogenous administration of rhGH results in a reduced secretion of phGH, the ratio between rhGH and phGH (“Rec/Pit ratio”) can provide evidence for doping with rhGH when compared to kit‐ and gender‐specific decision limits. According to current WADA guidelines [10], one of the two available kits “1” and “2” can be used as ITP. Each kit comprises a different set of “Rec” and “Pit” immunoassays based on alternative capture antibodies for either rhGH or phGH [9]. For detection purposes, the same antibody binding both rhGH and phGH is used in all four assays. In case of a presumptive adverse analytical finding (PAAF), the other kit has to be utilized for confirmation purposes [10].

As it was demonstrated that these immunoassays can be incompatible with LAGHs characterized by significant structural modifications such as somatrogon [8], it was of utmost importance to evaluate the detectability of redalsomatropin alfa.

#### 3.1.1 Cross‐Reactivity Test

First, the cross‐reactivity of redalsomatropin alfa with the anti‐GH antibodies employed in the Rec and Pit assays of the hGH luminescence immunoassay (LIA) Kits 1 and 2 was evaluated at three different analyte concentrations (3, 30, and 300 ng/mL). The results are summarized in Table 2. Analogous to somatrogon, the capture antibody for 22‐kDa hGH in Kit 1 was found to poorly bind to redalsomatropin alfa as reflected by estimated GH concentrations of 0.001 (< LOQ), 0.006 (< LOQ), and 0.062 ng/mL for 3, 30, and 300 ng/mL, resulting in a mean cross‐reactivity of 0.025%. It can be assumed that this is primarily due to the protection of the C‐terminal epitope of the antibody by the attached albumin. However, it has also to be considered that redalsomatropin alfa (~90 kDa) has a significantly higher MW than 22‐kDa hGH, resulting in a lower molarity (at least by a factor of 4) at a defined weight‐based drug concentration.

**TABLE 2:** Cross‐reactivity of redalsomatropin alfa with hGH immunoassays routinely employed in sports drug testing.

Spiked concentration	Found [ng/mL] (rate)			
	GH isoform Kit 1		GH isoform Kit 2	
	Rec	Pit	Rec	Pit
3 ng/mL	< LOQ (0%)	0.117 (3.90%)	0.370 (12.33%)	0.130 (4.33%)
30 ng/mL	< LOQ (0%)	1.265 (4.22%)	3.871 (12.90%)	1.520 (5.07%)
300 ng/mL	0.062 (0.02%)	14.210 (4.74%)	39.385 (13.13%)	14.287 (4.76%)

*Note:* Rate (%) = (measured value/target value) × 100.

By contrast, the Rec assay of Kit 2 yielded a mean cross‐reactivity of 12.8% with GH concentrations determined for the sheep serum samples fortified with 3, 30, and 300 ng/mL of 0.37 ng/mL (≡ 12.33%), 3.87 ng/mL (≡ 12.90%), and 39.39 ng/mL (≡ 13.13%). As the observed difference between the measured and actual drug concentrations is significantly higher than a factor of 4, it can be assumed that the large C‐terminal HSA also interferes with the epitope of this capture antibody.

For the anti‐phGH antibodies utilized in Kits 1 and 2, mean cross‐reactivities of 4.3% and 4.7% were observed. According to Bidlingmaier et al. [9], the recovery of 22‐kDa hGH with both Pit assays is only 39% and 34%, indicating that the employed antibodies are generally less efficient in GH binding. But also with an assumed difference of factor 4 due to the higher MW of redalsomatropin alfa, the measured values are significantly lower as expected. Therefore, it appears reasonable that the presence of the large HSA at the C‐terminus of 22‐kDa hGH in redalsomatropin alfa either equally reduces binding of the two anti‐phGH capture antibodies to the molecule, or—more likely due to the almost identical recovery determined with both kits—the interaction with the detection antibody, which is identical in all four immunoassays [9].

#### 3.1.2 Analysis of Serum Samples Fortified With Redalsomatropin Alfa

As no authentic post‐administration serum samples are available, the applicability of the GH isoform test to the detection of redalsomatropin alfa was further evaluated by analyzing three different sets of serum samples fortified with concentrations of 3 (*n* = 10), 30 (*n* = 10), and 300 ng/mL (*n* = 20). These samples were previously tested negative for rhGH with either Kit 1 or 2 (Table 3A,B). With Kit 1 (Table 3A), the Rec/Pit ratios were found to decrease with increasing concentrations of redalsomatropin alfa. This can be explained by the fact that only the Pit assay of this Kit was found to recognize the fusion protein. Consequently, all ratios were clearly below the thresholds for female and male athletes of 1.63 and 1.84. By contrast, the Rec/Pit ratios determined with Kit 2 (Table 3B) significantly increased with the amounts of redalsomatropin alfa present in the samples: The mean ratios of the female samples fortified with 3, 30, and 300 ng/mL were 0.71, 1.24, and 2.39, and for the male specimens, values of 1.03, 2.02, and 2.28 were determined. These findings show that samples containing high drug concentrations would produce suspicious test results with this kit; however, due to the reduced cross‐reactivity described in Section 3.1.1, the sensitivity/efficacy of this assay is significantly decreased. For instance, four out of five female and one out of five male samples fortified with redalsomatropin alfa at a concentration of 30 ng/mL yielded Rec/Pit ratios below the respective thresholds.

**TABLE 3:** Analysis of serum samples fortified with redalsomatropin alfa at concentrations of 3, 30 and 300 ng/mL with GH isoform differential immunoassays Kit 1 (A) and 2 (B).

Sample ID	(A) Rec/Pit ratio: Kit 1				(B) Rec/Pit ratio: Kit 2			
	Blank	3 ng/mL	30 ng/mL	300 ng/mL	Blank	3 ng/mL	30 ng/mL	300 ng/mL
F1	NA	0.306	NA	0.024	0.325	0.525	NA	2.243
F2	NA	0.384	NA	0.014	0.576	1.063	NA	2.288
F3	NA	0.522	NA	0.051	0.476	0.689	NA	2.622
F4	NA	0.537	NA	0.036	0.513	0.760	NA	2.624
F5	0.430	0.437	NA	0.035	NA	0.489	NA	2.849
F6	0.788	NA	0.544	0.119	NA	NA	1.421	2.682
F7	0.704	NA	0.180	0.024	NA	NA	2.081	2.480
F8	0.444	NA	0.362	0.100	NA	NA	1.005	2.182
F9	0.618	NA	0.460	0.114	NA	NA	1.058	2.160
F10	0.510	NA	0.466	0.206	NA	NA	0.647	1.797
M1	0.637	0.299	NA	0.010	NA	1.241	NA	2.157
M2	NA	0.452	NA	0.073	0.473	0.553	NA	2.025
M3	NA	0.227	NA	0.012	0.308	1.009	NA	2.251
M4	NA	0.287	NA	0.013	0.409	0.935	NA	2.417
M5	NA	0.359	NA	0.013	0.534	1.404	NA	2.309
M6	NA	NA	0.060	0.012	0.130	NA	1.753	2.183
M7	NA	NA	0.087	0.013	0.523	NA	2.099	2.300
M8	NA	NA	0.077	0.013	0.379	NA	2.033	2.153
M9	NA	NA	0.059	0.011	0.474	NA	2.152	2.401
M10	NA	NA	0.064	0.013	0.339	NA	2.052	2.561
Mean F	0.582	0.437	0.402	0.072	0.472	0.705	1.242	2.393
Mean M	0.637	0.325	0.069	0.018	0.397	1.028	2.018	2.276

*Note:* NA = samples with the respective drug concentration were not available due to a limited sample volume.

So overall, the results of both experiments demonstrate that the detectability of redalsomatropin alfa with the GH isoform test is significantly impaired as only Kit 2 can detect the presence of the drug and only at high concentrations. In clinical trials with redalsomatropin alfa, mean maximum plasma concentrations of 545, 1309 and 3595 ng/mL were measured for single doses of 0.25, 0.5, and 1 mg/kg^4^. With an elimination half‐life of 29–43 h, it can be assumed that samples collected during the first days following injection would be tested positive with Kit 2, but unfortunately, a confirmation with Kit 1 would not be possible. When Kit 1 is used as ITP, doping with redalsomatropin alfa would potentially remain undetected. Therefore, the implementation of the drug into the existing mass spectrometric detection assay for somatrogon [8] was of utmost importance—not only to reanalyze samples characterized by unusually low Rec/Pit ratios measured with Kit 1 and high Rec/Pit ratios when analyzed with Kit 2, but also to generally allow for a more sensitive testing for redalsomatropin alfa.

### 3.2 Mass Spectrometric Detection of Redalsomatropin Alfa

**FIGURE 2:**
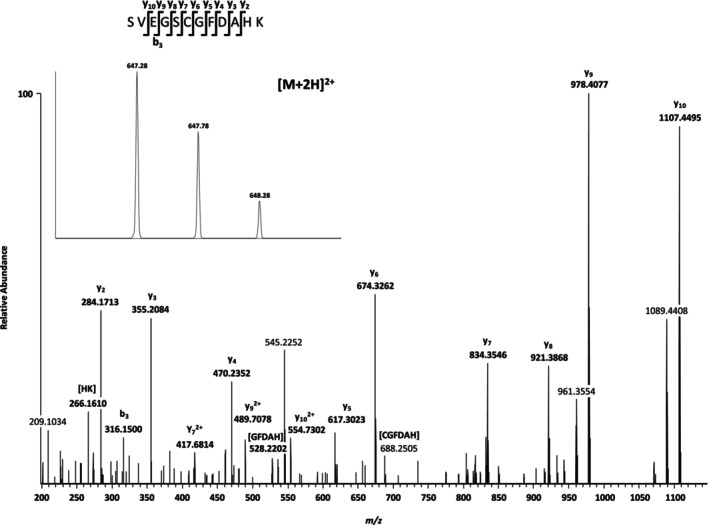
HRMS and MS/MS spectra of the diagnostic peptide T_21_ (*m/z* = 647.28, SVEGSCGFDAHK) recorded on an Orbitrap Exploris 480 interfaced with a Vanquish UHPLC.

**FIGURE 3:**
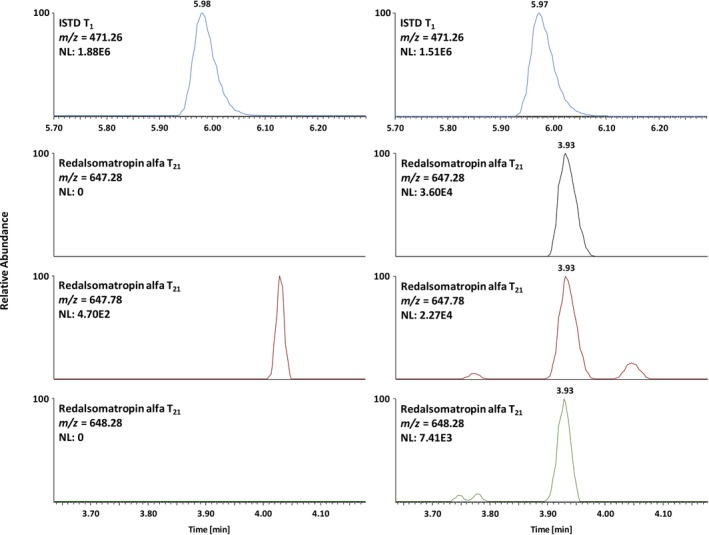
Exemplary extracted ion chromatograms (mass tolerance ± 5 ppm) of the three most abundant isotopes of peptide T_21_ (*m/z* = 647.28, 647.78, and 648.28) diagnostic for redalsomatropin alfa and the ISTD peptide T_1_ (*m/z* = 471.26) in a blank and a serum sample fortified with 100 ng/mL of the drug.

As redalsomatropin alfa represents an artificial fusion protein composed of 22‐kDa hGH and modified HSA [4], two different tryptic peptides can theoretically be employed for its unambiguous mass spectrometric identification: Peptide T_21_ comprises the C‐terminus of 22‐kDa hGH and the N‐terminus of modified HSA, and its sequence does therefore not naturally occur in the human body as it was confirmed by a Protein BLAST database search. The detectability of this peptide could be verified from an in‐solution tryptic digest of redalsomatropin alfa reference material, and the corresponding HRMS and MS/MS spectra are displayed in Figure 2. The second peptide T_63_ is located in the albumin domain and includes an amino acid exchange (A → T) at position 511 [4], which also results in a sequence specific for redalsomatropin alfa. Unfortunately, this peptide could not be detected from the in‐solution digest, which can probably be attributed to the N‐glycosylation present at the asparagine residue at position 509. Therefore, only peptide T_21_ was included in the existing LC‐HRMS/MS method for somatrogon and 22‐kDa hGH, and the modified approach was validated according to current WADA guidelines [14]. As shown in Table 4, the method was found to be highly specific and reliable at a concentration of 100 ng/mL. The LOD was estimated as 50 ng/mL, and the approach was linear from 50 to 1000 ng/mL. Figure 3 shows the extracted ion chromatograms of a blank and a serum specimen fortified with redalsomatropin alfa at a concentration of 100 ng/mL. The target peptide T_21_ was unambiguously detected, and no interference was present in the blank specimen. The use of plasma instead of serum (robustness) had no effect on the method's performance when analyzing samples fortified with 100 ng/mL of the drug. At a concentration of 1000 ng/mL, no sample carryover was observed for both the LC‐HRMS/MS measurements and magnetic beads reusage. Sample extracts were stable for at least 5 days when stored in the autosampler at 10°C, and the recovery was estimated as approximately 20%. This is significantly lower than the value of ~45% determined for somatrogon in a previous study [8]. According to the literature [4, 15], both redalsomatropin and somatrogon are characterized by a lower binding affinity to the GH when compared to hGH. As the attachment of a large molecule such as HSA has a higher impact on the structure of 22‐kDa hGH than the attachment of the CTP of human β‐hCG, a lower recovery appears to be plausible.

**TABLE 4:** Results of method validation.

Validation parameter	*n*	Concentration(s) [ng/mL]	Redalsomatropin alfa
Selectivity	10	——	0/10
Reliability of detection	10	100	10/10
LOD	6	25	3/6
	6	50	6/6
	6	75	6/6
Linearity	7	50–1000	*R* ^2^ = 0.984
Robustness (plasma)	10	100	10/10
Carryover (magnetic beads)	1	1000	No
Carryover (LC‐HRMS/MS)	1	1000	No
Sample extract stability	7	50–1000	≥ 5 days
Recovery (magnetic beads)	4	250	21%

Overall, the acquired data could demonstrate that the modified method is fit for purpose as ITP and authentic post‐administration samples should be analyzed as proof of concept as soon as possible. Moreover, a full confirmation protocol involving targeted MS^2^ measurements to evaluate multiple diagnostic product ions and ion ratio stabilities will be the subject of future studies.

## 4 Conclusions

Redalsomatropin alfa (JR‐142) is an emerging LAGH whose misuse in sports is prohibited at all times due to the presumed anabolic and lipolytic effects. Within this research project, its detectability with the hGH isoform differential immunoassays currently employed by anti‐doping laboratories for rhGH routine testing was evaluated, and similar to somatrogon, a fusion protein of 22‐kDa hGH and the CTP of β‐hCG, the structural modifications were found to interfere with antibody binding: Only Kit 2 could recognize the drug, and while its cross‐reactivity with somatrogon was similar to native 22‐kDa hGH, the compatibility with redalsomatropin alfa was clearly reduced, resulting in a significantly lower sensitivity. Therefore, an alternative testing strategy had to be developed, and as *in silico* tryptic digestion yielded an artificial fusion peptide comprising the C‐terminus of 22‐kDa hGH and N‐terminus of HSA suitable for an unambiguous mass spectrometric identification of the drug, it was implemented into the existing detection assay for the GH analogue somatrogon. This approach is based on affinity purification with GHR‐Fc‐conjugated magnetic beads, tryptic digestion, and LC‐HRMS/MS and was found to allow for a specific and sensitive detection of redalsomatropin alfa. Moreover, these data show that the assay can further be modified to include other LAGHs and thus ensure the detectability of such compounds in doping control serum samples.

As clinical studies [4] demonstrated significant effects on IGF‐I levels, future research projects should also investigate the applicability of the hGH biomarker test to detect the misuse of redalsomatropin alfa in sports.

## Funding

This work was supported by the National Anti‐Doping Agency (NADA, Bonn, Germany), Sport Ireland (Dublin, Ireland), the Federal Chancellery of the Federal Republic of Germany (Berlin, Germany), and the Manfred‐Donike Institute for Doping Analysis (Cologne, Germany).

## Conflicts of Interest

The authors declare no conflicts of interest.

## Data Availability

The data that support the findings of this study are available from the corresponding author upon reasonable request.
